# Separating Bedtime Rest from Activity Using Waist or Wrist-Worn Accelerometers in Youth

**DOI:** 10.1371/journal.pone.0092512

**Published:** 2014-04-11

**Authors:** Dustin J. Tracy, Zhiyi Xu, Leena Choi, Sari Acra, Kong Y. Chen, Maciej S. Buchowski

**Affiliations:** 1 Energy Balance Laboratory, Division of Gastroenterology, Hepatology and Nutrition, Department of Medicine, Vanderbilt University, Nashville, Tennessee, United States of America; 2 Department of Biostatistics, Vanderbilt University Medical Center, Nashville, Tennessee, United States of America; 3 Department of Pediatrics, Vanderbilt University Medical Center, Nashville, Tennessee, United States of America; 4 National Institute of Diabetes and Digestive and Kidney Disease, Diabetes, Endocrinology, and Obesity Branch, National Institutes of Health, Bethesda, Maryland, United States of America; Cardiff University, United Kingdom

## Abstract

Recent interest in sedentary behavior and technological advances expanded use of watch-size accelerometers for continuous monitoring of physical activity (PA) over extended periods (e.g., 24 h/day for 1 week) in studies conducted in natural living environment. This approach necessitates the development of new methods separating bedtime rest and activity periods from the accelerometer recordings. The goal of this study was to develop a decision tree with acceptable accuracy for separating bedtime rest from activity in youth using accelerometer placed on waist or wrist. Minute-by-minute accelerometry data were collected from 81 youth (10–18 years old, 47 females) during a monitored 24-h stay in a whole-room indirect calorimeter equipped with a force platform covering the floor to detect movement. Receiver Operating Characteristic (ROC) curve analysis was used to determine the accelerometer cut points for rest and activity. To examine the classification differences, the accelerometer bedtime rest and activity classified by the algorithm in the development group (n = 41) were compared with actual bedtime rest and activity classification obtained from the room calorimeter-measured metabolic rate and movement data. The selected optimal bedtime rest cut points were 20 and 250 counts/min for the waist- and the wrist-worn accelerometer, respectively. The selected optimal activity cut points were 500 and 3,000 counts/min for waist and wrist-worn accelerometers, respectively. Bedtime rest and activity were correctly classified by the algorithm in the validation group (n = 40) by both waist- (sensitivity: 0.983, specificity: 0.946, area under ROC curve: 0. 872) and wrist-worn (0.999, 0.980 and 0.943) accelerometers. The decision tree classified bedtime rest correctly with higher accuracy than commonly used automated algorithm for both waist- and wrist-warn accelerometer (all p<0.001). We concluded that cut points developed and validated for waist- and wrist-worn uniaxial accelerometer have a good power for accurate separation of time spent in bedtime rest from activity in youth.

## Introduction

Accelerometry has been frequently used for the measurement of time spent in activities performed at various intensities and for the prediction of physical activity (PA) related energy expenditure [1]–[4]. Accelerometer applications range from clinical interventional trials [5], [6] to epidemiological studies [7]–[9]. Recent technological advances such as watch size devices with high data storage capacity allows capturing PA for extended monitoring period (e.g. 24 hours per day for 7 days). This increasingly popular “24/7”approach can lead to more detailed assessments of individual's PA amount and patterns, which is particularly relevant because of the rapidly growing interest in sedentary behavior and sleep patterns and their relationship to health in children and adolescents [10]–[13].

The first step in the 24-hour accelerometry data analysis is assessing a person's compliance with the monitor wearing instructions using a wearing/nonwearing algorithm such as proposed by Choi et al. [14] or Troiano et al. [7]. The next step is to discriminate bedtime rest from activity that include sedentary behaviors and activity usually classified as light, moderate, and vigorous PA intensity categories [15]. Traditionally, activity time is scored using information from a self-report [16], or more objectively using signals from accelerometers equipped with a light sensor, an inclinometer, or an event button. Validity and limitations of these methods were described elsewhere [17].

An alternative approach is to use an automated algorithm that classifies accelerometer wear time into the bedtime rest and activity categories using a decision tree that uses empirically determined cut points from the accelerometer output, i.e. counts. These procedures include scoring algorithms for assessing sleep such as a device-specific algorithm developed in a group of adults (n = 20) and children (n = 16) by Sadeh et al. using polysomnography (PSG) as a reference standard [18]. A similar algorithm for assessing sleep using Actigraph GT1M accelerometer was developed in a group of 15 children (10–11 years old) using sleep diaries and another accelerometer as a reference standard [19]. Recently, Wrzus and colleagues have proposed an algorithm to identify sleep in a natural environment based on body posture classification [20]. The strength and limitation of using accelerometry to assess sleep have been described and summarized elsewhere [21]. These algorithms, however, were developed specifically to assess sleep rather than bedtime rest which might include other than sleep forms of bedtime rest such as lying and viewing television or short naps. Further improvements to the existing algorithms are needed to automate the determination of bedtime rest and activity from the accelerometry data. These new or improved algorithm(s) should be applicable to clinical and epidemiologic studies conducted in various populations assess physical activity using accelerometry.

Thus, we hypothesized that the Actigraph monitor placed on waist or wrist will correctly categorize bedtime rest from activity (wake) in comparison with data from the whole-room indirect calorimeter. The primary goal of this study was to develop and validate a decision tree for classification of bedtime rest and activity intervals from Actigraph accelerometer counts and compare its sensitivity and specificity with the actual bedtime rest or active status determined by a whole-room indirect calorimeter. The accuracy of the developed decision tree was assessed by comparing with commonly used Sadeh's algorithm to predict sleep and activity from 24-h accelerometry data [18], [22], [23]. The second goal was to assess the effect of the monitor placement (waist versus wrist) on valid bedtime rest and activity classification without using complementary assessment methods (e.g., sleep diaries) in youth. These two locations (waist and/or wrist) are the most commonly used for an accelerometer placement in epidemiological and clinical studies designed to measure the amount and patterns of physical activity in a specific study population including youth.

## Materials and Methods

All applicable institutional and governmental regulations concerning the ethical use of human volunteers were followed during this research, in accordance with the ethical principles of the Helsinki-II Declaration. The study protocol was approved by the Vanderbilt University Medical Center Institutional Review Board. Before the study, participating youth and their parents or guardians signed a printed informed consent or assent.

### Participants

Young healthy volunteers (age 10 to 17 years) were recruited from the Nashville, Tennessee area using flyers, email distribution, and word of mouth to a prospective study focused on methodological aspects of PA measurement in youth [24]. In the recruitment process, sex (male vs. female), race (white vs. black), age (categories), and BMI (categories) were balanced. All applicable institutional and governmental regulations concerning the ethical use of human volunteers were followed during this research, in accordance with the ethical principles of the Helsinki-II Declaration. Before the study, participating youth and their parents or guardians signed an informed consent or assent, which was approved by the Vanderbilt University Medical Center Institutional Review Board.

### Study design and protocol

Study participants (n = 81) spent approximately 24-hours in a whole-room indirect calorimeter [25] and followed a structured protocol designed for simultaneous measurements of PA and energy expenditure. During the entire room calorimeter stay, each participant wore Actigraph accelerometers mounted on their waist on the dominant side and dominant wrist. The room calorimeter is an airtight room with windows and an airtight pass and is equipped with a toilet, sink, desk, chair, telephone, multimedia, and exercise equipment. The room assures high-precision measurements in a controlled environment and semi-naturalistic conditions (i.e. not wearing a breathing mask), and it allows for adjusting the intensity of any PA task to the individual's capability. The force platform covering the entire living area inside the room calorimeter is supported by precision force transducers. The force platform allowed computer-aided measurement (60 times per second) of body position, displacement, and mechanical forces with an accuracy of 97% or higher. Technical details of the indirect calorimeter measurement approach have been reported previously [25]. The protocol inside the room included a broad range of activities ranging from light and sedentary tasks such as eating meals and snacks and self-care activities to moderate and vigorous PA, such as stretching, jogging and walking on a treadmill, biking on a stationary bike. During the remaining time (∼5 hours) when no activity was specifically scheduled, participants were asked to engage in their normal daily routine as much as possible without specific suggestions. They were instructed to start a bedtime rest at 10:00 pm and were woken up at 6:00 am. Bedtime rest was defined as the time spent on a mattress bed when no significant movement was detected by the force platform. Participants also recorded their activities in a diary with a detailed schedule, reporting any episodes of accidental monitor non-wear intervals and other relevant comments. The study staff reviewed the diary with each participant after finishing the study. The data was not used for bedtime rest assessment. Complete data sets were available for 81 participants and used in the algorithm development and validation.

### Measures

#### Physical activity (PA)

PA was measured by two Actigraph GT1M accelerometers (ActiGraph, Pensacola, FL), placed on the anterior axillary line on the dominant side of the waist and the dominant wrist. The GT1M monitor (firmware version 6.2.0) is a self-calibrated uniaxial accelerometer that is generates data in counts per user-defined epoch length [22], [26]. In this study, acceleration data were collected at a 1-sec epoch and summed as counts per minute to facilitate time synchronization with recordings from the room calorimeter and force platform. ActiLife software version 4.4.1 was used for initializing the monitors and downloading the data [22].

#### Movement-related mechanical work (Watt/min)

Mechanical work was measured using a force platform covering the room calorimeter floor that has been shown to measure accurately and reproducibly horizontal and vertical mechanical work and was sensitive to pressure changes caused by a person's movement during activity and bedtime rest [27].

#### Energy Expenditure (kcal/min)

Energy expenditure during the entire period (∼24-h) was calculated minute-by-minute from measured rates of oxygen (O_2_) consumption and carbon dioxide (CO_2_) production using Weir's equation [28]. The accuracy of our metabolic chamber for measuring energy expenditure by routine alcohol combustion tests was 99.2±0.5% (mean ± SD) over 24 hours and 98.6±2.1% over 30 minutes [25]. The system detects reliably short-term changes in metabolic rate to 2.7% over 30 min and 0.6% over 2 h measurement period.

The resolution of the indirect calorimetry was 2 min and was extrapolated to 1 min using an automated algorithm [29].

#### Room calorimeter measured bedtime rest and activity intervals

Room calorimeter-measured energy expenditure and the force platform-measured mechanical work threshold values and plots were used to classify activity minute-by-minute into bedtime rest or activity. The classification was performed using an automatic computer algorithm and verified independently by two experts in the whole-room indirect calorimeter data analysis (MSB and LC). Time periods (minutes) for which the difference between computer classification and the experts consensus was found, the experts' assessment was used as an indicator of bedtime rest or activity. The high degree of agreement was observed for bedtime rest periods estimate between the algorithm and experts. The classification was rendered as a binary indicator variable and synchronized minute-by-minute with the accelerometer recordings data taking under consideration a 2-min time lag between room calorimeter and accelerometry. We found this approach more accurate than information extracted from self-reports. The study protocol did not include video recording because of concerns related to children's privacy during the 24 h room calorimeter stay expressed by children and parents in our previous studies.

### Accelerometry data decision tree development

#### Selecting criterion values

The key criterion values used in the decision tree development were cut points for bedtime rest and activity and time windows. The cut points were generated separately for waist and wrist worn accelerometers using the receiver operation curve (ROC) procedure for the determination of the threshold with optimal sensitivity and specificity [30].

*Threshold average* was defined as a number of counts/min in any 1-hour time block during the entire monitoring period.

*Bedtime rest cut point* was defined as the maximum number of counts/min allowed for bedtime rest in any 1-hour block. The cut-point was set initially at 50 counts/min (CP_0_), which was previously used as a rest cut-point for waist-worn Actigraph model 7164 monitor [31].

*Activity cut point* was defined as the minimum number of counts per minute needed for the activity.

*Window* was defined as an optimal period searched for the bedtime rest starting and ending points and was set at 60 min [14].

#### Decision tree development for analysis of minute-by-minute accelerometer data

**Figure 1:**
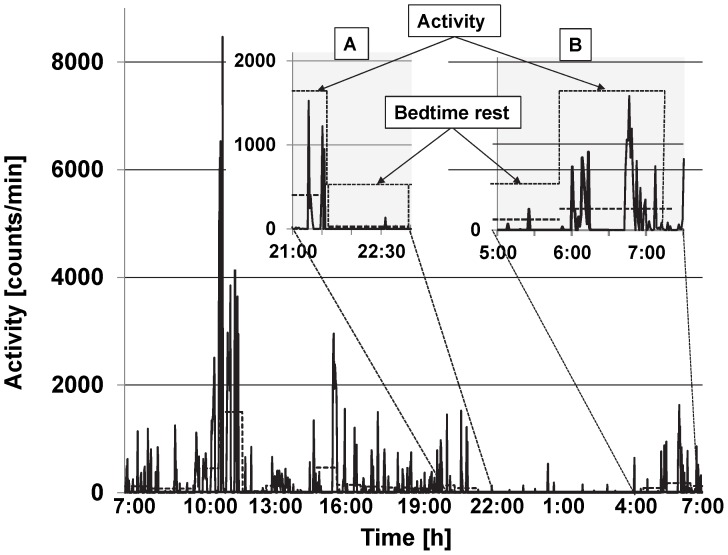
Representative data plot for one participant (17 years old male) from a 24-h stay in the room calorimeter. The solid line represents Actigraph recordings (counts/min), and the thick horizontal dash line represents average counts/hour. The insets are representative periods in which transition from activity to bedtime rest (A) and from bedtime rest to activity (B) occurred.

**Figure 2:**
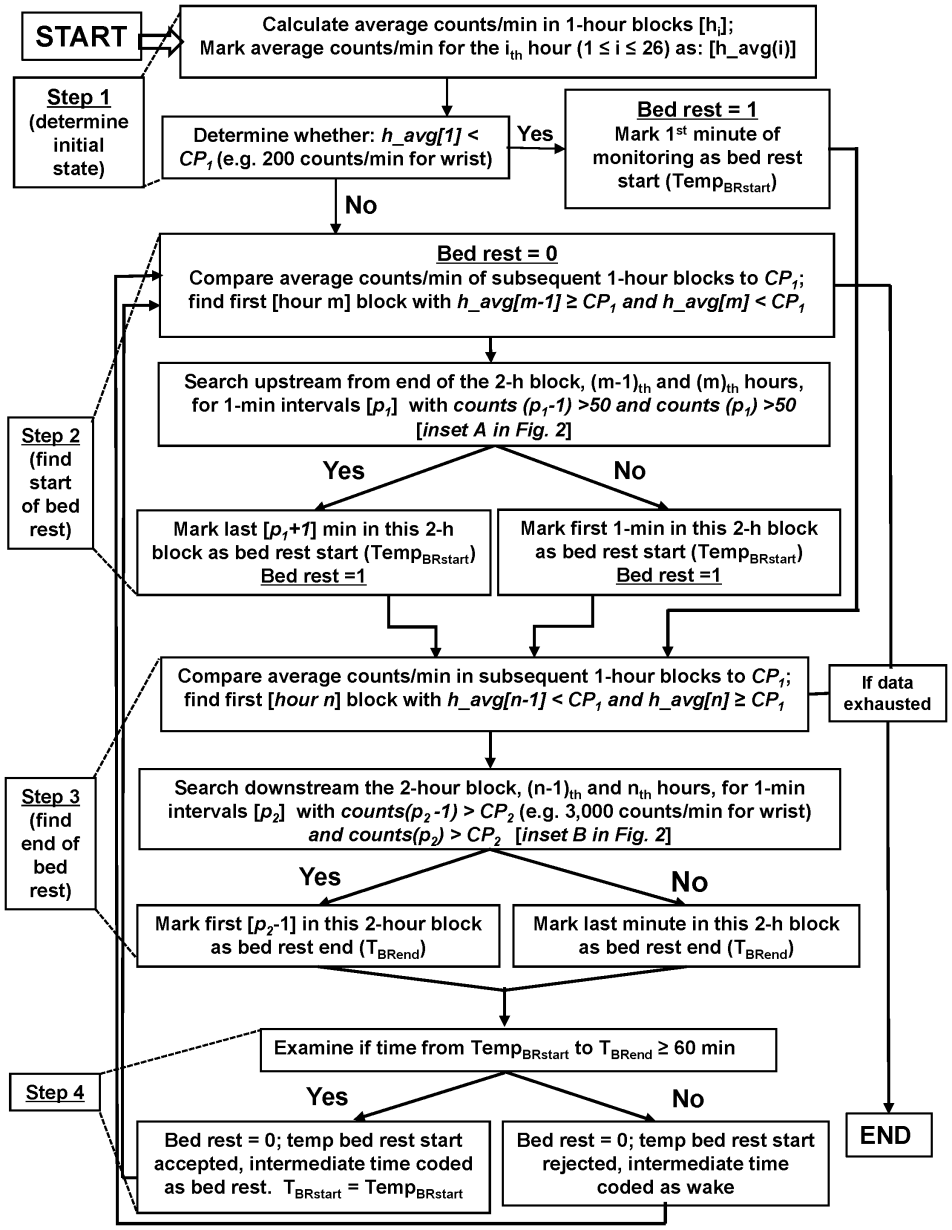
The decision tree for the classification of bedtime rest and activity accelerometer recordings. The decision tree algorithm was using various sets of cut points for waist and wrist worn accelerometers.

An automated decision tree was developed from manually marked graphed data for the bedtime rest when there was no other reliable measure. The tree was designed to find blocks at least 1-hour long with a low average activity level (less than 50 counts/min) and sporadic very short periods of activity. The beginnings and ends of these blocks were marked by transitions to sustained levels of higher activity, as shown in Figure 1. Averaging across 1-hour blocks attenuated the effect any spike in activity, while efficiently processing data. The logic behind the rules was to find periods with minimal activity. Using these rules, a four stages decision tree was developed (Figure 2). In Stage 1, the algorithm calculates the average counts/min for each 1-hour time block for the entire monitoring period (e.g., 24 h). Next, the algorithm determines if the counts/min average in the first 1-hour block is lower, equal to, or higher than the tested lower bedtime rest cut point (CP_1_) value (Table 1). If the block average is lower than CP_1_ (e.g. 200 counts/min for wrist), the algorithm marks the 1^st^ minute as a temporary beginning of bedtime rest (Temp_BRstart_) and proceeds to Stage 3. If the block average is equal to or higher than CP_1_, the algorithm classifies the block as activity and proceeds to Stage 2.

**Table 1:** Characteristics of study participants.

	All participants (n = 81)	Development Sample (n = 40)	Validation Sample (n = 41)	p value^a^
Age (years)	13.44±2.19 (10 to 17)	13.23±2.15 (10 to 17)	13.66±2.23 (10 to 17)	0.38
Height (m)	1.61±0.09 (1.39 to 1.87)	1.60±0.10 (1.39 to 1.79)	1.62±0.09 (1.43 to 1.87)	0.69
Weight (kg)	67.48±19.47 (38.6 to 129.5)	66.60±19.13 (38.8 to 129.5)	68.33±20.00 (38.6 to 125.7)	0.36
Body mass index [BMI] (kg/m^2^)	25.78±5.93 (16.32 to 44.03)	25.77±5.85 (16.32 to 44.03)	25.79±6.07 (16.40 to 38.54)	0.98
BMI percentile^b^	83.81±20.75 (5.48 to 99.80)	85.56±18.77 (14.69 to 99.80)	82.11±22.62 (5.48 to 99.59)	0.46
*Sex*				
Female	47	22	25	
Male	34	18	16	
*Ethnicity*				
African American	22	10	12	
White	58	30	28	
Hispanic	1	0	1	

Values are presented as mean ± standard deviation and (range).

a -two-sample t-test,

b ^-^BMI percentile – Body Mass Index (BMI) percentile calculated from the Centers for Disease Control (CDC) BMI-for-age growth charts.

Stage 2 and 3 determine beginning or end of each bedtime rest period. The algorithm initially searches the non-rolling 1-h averages to identify a window in which the change occurred and then searches minute-by-minute through the identified window to detect the minute the change occurred. In Stage 2, the algorithm compares the remaining 1-h blocks average counts/min calculated in Stage 1 to CP_1_ and searches for a 2-hour block in which 1^st^ hour average is equal or higher than CP_1_ and the 2^nd^ hour average is lower than CP_1_. After finding a 2-h block, the algorithm searches upstream from the block's end for the first two consecutive 1-min intervals with counts higher than CP_0_ (50 counts/min). The 1^st^ minute after the 2^nd^ minute of the interval marks the beginning of bedtime rest (Temp_Rstart_). If no two consecutive 1-min intervals higher than CP_0_ are found, the 1^st^ min of the 2-hour block is marked as the beginning of the bedtime rest. The algorithm allows for the theoretical possibility that the condition (i.e., counts (p_1_-1)>50 and counts (p_1_)>50) is never met and creates an additional box in the flow chart. Both boxes find a start time of the bedtime rest but use different methods to achieve this goal. The “left” box marks the beginning of the bedtime rest from a block meeting a condition (p_1_-1<50 and p_1_<50). If this procedure should fail, the “right” box marks the 1^st^ min of the block as a start time of the bedtime rest. In Stage 3, the algorithm compares the average counts/min in subsequent 1-h blocks to CP**_1_** until it finds first 2-hour block in which 1^st^ hour counts/min average is lower than CP_1_ and the 2^nd^ hour count average is equal or higher than CP_1_. When the block is identified, the algorithm searches the block downstream for first two consecutive 1-min intervals with counts/min higher than CP_2_ (e.g. 3,000 counts/min for wrist). The minute before the 1^st^ minute of the 2-min interval is marked as a temporary bedtime rest end (T_BRend_). If no such 1-min intervals are found, the last minute of the 2-hour block is marked as a bedtime rest end (T_BRend_).

In Stage 4, the algorithm examines the length of the temporary bedtime rest period. If the period is shorter than 1 hour, the temporary bedtime rest end (Temp_BRstart_) is discarded, and the period is marked as activity. If the bedtime rest is equal or longer than 1 hour, this period is marked as bedtime rest. The next minute (T_BRend+1_) is marked as activity, and the algorithm repeats Stage 2 with the remainder of the data in the dataset.

#### Commonly used computer algorithm

Automated computer algorithm originally developed by Sadeh [18] was used to assess bedtime rest. Minute-by-minute accelerometry data were rendered as a binary indicator of “sleep” or “wake” [23].

### Data analysis

The subjects were allocated to developmental and validation groups using the coin flipper feature on publicly available website Random.org. For each participant in the development group, the entire monitoring period (∼24 h) classified by the decision tree algorithm, as either bedtime rest or activity was compared minute-by-minute to the bedtime rest or active status obtained from the room calorimeter. Time intervals (minutes) different from the corresponding room calorimeter value (0 vs.1 or 1 vs. 0) were categorized as false positive or false negative. The Receiver Operating Characteristic (ROC) curves were plotted with various cut points as a function of sensitivity and specificity in discriminating bedtime rest from activity. Sensitivity was defined as the probability of correctly classifying bedtime rest period and specificity was defined as the probability of correctly classifying activity period based on the room calorimeter-determined rest and activity status. Specificity and sensitivity were considered equally important. The area under each cut point (AUC) in the ROC curve is the product of sensitivity and specificity. The AUC was used to evaluate overall performance of each algorithm threshold condition. For each combination of two cut points, medians of sensitivity, specificity, and the AUC were calculated across subjects in the development data set. The medians were used to plot the ROC curves. The optimal cut points obtained from the development set were used in testing of the validation set. The 2-fold validation method was used based on a relatively large sample size (n = 40 and 41) in the development and validation sets, respectively. The final data set and program are available from the corresponding author (MSB) upon request.

### Statistical analysis

Wilcoxon signed rank test was used to test the difference in the indices between selected threshold conditions. Data from waist and wrist accelerometers were analyzed separately. Bedtime rest calculated using decision tree and commonly used algorithm (Sadeh's) were compared using the Wilcoxon signed rank test since the outcomes distributions were skewed. Results are presented as means or medians, standard deviations (SD), and ranges. The programming language R version 2.13.0 [32] was used to develop and implement the decision tree and algorithms and to perform the statistical analyses.

## Results

### Participants' characteristics

There were no differences (all P>0.05) in personal characteristics between the development and validation sets (Table 1).

### Decision tree parameters

A representative plot illustrating accelerometry recordings from the 24-stay in the room calorimeter is presented in Figure 1.

#### Bedtime rest and activity cut points for the waist-worn accelerometer

**Figure 3:**
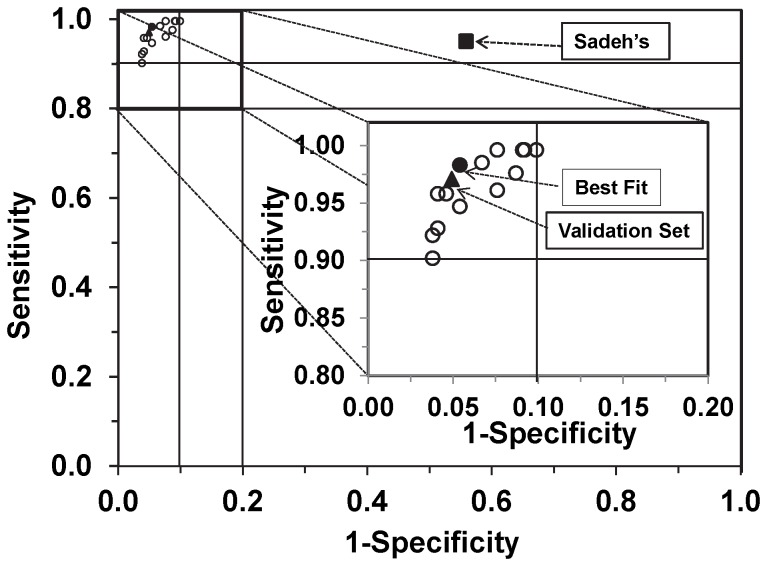
Waist-worn accelerometer data for the development group showing tradeoff between sensitivity and specificity. Each circle represents sensitivity (y-axis) and 1 – specificity (x-axis), calculated using ROC analysis for a curve (not shown) of a respective set of cut points. The solid circle [•] in the inset represents the selected optimal cut points (counts/min) for bedtime rest (CP_1_) and activity (CP_2_). The corresponding values are in Table 2 (bold). The solid square [▪] represents Sadeh's algorithm (Table 3) and the solid triangle [▴] represents the validation set (Table 4).

The area under curve for the ROC curves drawn using the medians of sensitivity and 1-specificity for bedtime rest cut points from 10 to 30 counts/min, and activity cut points from 400 to 600 counts/min are in Figure 3. The bedtime rest cut points from 20 to 30 counts/min in combination with activity cut points 400 or 500 counts/min resulted in the median AUC>0.8 and the medians of sensitivity and specificity >0.88 (Table 2).

**Table 2:** Medians for the area under curve (AUC), sensitivity, and specificity for various cut points (counts/min) tested in the development sample set using Receiver Operating Characteristic (ROC) curves for accelerometer worn a waist or wrist during a ∼24-h stay in a whole-room indirect calorimeter.

Waist					Wrist				
CP_1_ (counts/min)	CP_2_ (counts/min)	AUC ± SD^a^	Sensitivity^b^	Specificity^c^	(CP_1_) (counts/min)	CP_2_ (counts/min)	AUC ± SD^a^	Sensitivity^b^	Specificity^c^
10	400	0.832±0.174	0.902	0.962	150	3500	0.898±0.078	0.969	0.965
10	500	0.847±0.175	0.922	0.962	175	2500	0.915±0.084	0.961	0.983
10	600	0.847±0.160	0.922	0.962	175	3000	0.922±0.077	0.976	0.983
15	400	0.859±0.149	0.928	0.959	175	3500	0.909±0.072	0.981	0.964
15	500	0.859±0.152	0.958	0.959	200	2500	0.920±0.080	0.976	0.983
15	600	0.860±0.148	0.958	0.954	200	3000	0.929±0.076	0.982	0.982
20	400	0.856±0.150	0.947	0.946	200	3500	0.928±0.071	0.991	0.964
**20**	**500**	**0.872**±**0.154**	**0.983**	**0.946**	225	2500	0.924±0.069	0.981	0.982
20	600	0.870±0.149	0.985	0.933	225	3000	0.929±0.065	0.997	0.980
25	400	0.852±0.150	0.961	0.924	225	3500	0.928±0.061	0.998	0.964
25	500	0.861±0.156	0.996	0.924	250	2500	0.943±0.066	0.996	0.980
25	600	0.855±0.152	0.996	0.908	**250**	**3000**	**0.943**±**0.063**	**0.999**	**0.980**
30	400	0.856±0.154	0.976	0.913	250	3500	0.939±0.066	1.000	0.964
30	500	0.856±0.157	0.996	0.909	275	2500	0.943±0.066	0.996	0.980
30	600	0.854±0.154	0.996	0.901	275	3000	0.934±0.061	1.000	0.978

Bolded values are optimal cut points for bedtime rest (CP_1_) and activity (CP_2_).

a - Area under the ROC curve calculated as sensitivity multiplied by specificity before data were rounded;

b - defined as the probability of correctly classifying bedtime rest period;

c - defined as the probability of correctly classifying activity period.

#### Bedtime rest and activity cut points for the wrist-worn accelerometer

**Figure 4:**
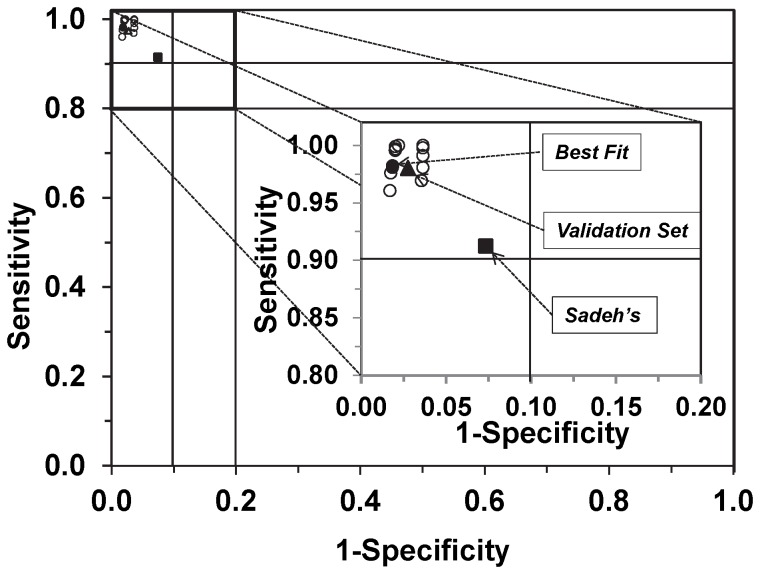
Wrist- worn accelerometer data for the development group showing tradeoff between sensitivity and specificity. Each circle represents sensitivity (y-axis) and 1 – specificity (x-axis), calculated using ROC analysis for a curve (not shown) of a respective set of cut points. The solid circle [•] in the inset represents the selected optimal cut points (counts/min) for bedtime rest (CP_1_) and activity (CP_2_). The corresponding values are in Table 2 (bold). The solid square [▪] represents Sadeh's algorithm (Table 3) and the solid triangle [▴] represents the validation set (Table 4).

The area under the ROC curves drawn using the medians of sensitivity and 1-specificity for bedtime rest cut points from 100 to 300 counts/min and activity cut points from 2,500 to 3,500 counts/min are in Figure 4. The bedtime rest cut points from 250 to 300 counts/min in combination with activity cut point 3,000 counts/min resulted in the median AUC>0.89 and the medians of sensitivity and specificity >0.94 (Table 2).

### Optimal cut points

Sensitivity increased and specificity decreased with the increase of the cut point values for both bedtime rest and activity. The optimal bedtime rest cut point (CP_1_) was 20 counts/min for the waist and 250 counts/min for the wrist-worn accelerometer. The optimal cut points for activity (CP_2_) were 400 and 3000 counts/min for the waist and the wrist-worn accelerometer, respectively.

### Comparison between development and validation sets

Sensitivity, specificity, and AUC were no different (all p>0.05) for both development and validation data sets for accelerometer worn either on the waist or wrist (Table 3).

**Table 3:** Comparison of bedtime rest classification from accelerometer placed on waist or wrist in the development and validation groups with classification obtained using whole- room indirect calorimeter.

Monitor placement	Group	AUC^a^	Sensitivity^b^	Specificity^c^	p-value^d^
Waist	Development^e^^,^ f	0.872	0.983	0.946	>0.05
	Validation^f^	0.859	0.968	0.968	
Wrist	Development^e^^,^ g	0.943	0.999	0.980	>0.05
	Validation^g^	0.923	0.975	0.967	

a – Area under the ROC curve calculated as sensitivity multiplied by specificity before data was rounded;

b - defined as the probability of correctly classifying bedtime rest period;

c - defined as a probability of correctly classifying activity period;

d - Wilcoxon signed rank test;

e - automated computer algorithm;

f - cut points were 20 counts/min (bedtime) and 500 counts/min (activity);

g - cut points were 250 counts/min (bedtime) and 3000 counts/min (activity).

### Comparison of the decision tree and commonly used algorithm (Sadeh's)

Accuracy of the decision tree to classify correctly bedtime rest was significantly higher than commonly used automated algorithm for both waist- worn (0.827 and 0.432, p<0.0001) and wrist-worn accelerometer (0.924 and 0.808, p<0.0001). The data are presented in Table 4.

**Table 4:** Comparison of bedtime rest classification from accelerometer placed on waist or wrist calculated using Sadeh's algorithm and the decision tree with classification obtained using whole- room indirect calorimeter.

Monitor placement	Bedtime rest assessment method	AUC^a^	Sensitivity^b^	Specificity^c^	p-value^d^
Waist	Algorithm (Sadeh)^e^	0.429	0.978	0.437	<0.001
	Decision tree^f^	0.859	0.983	0.946	
Wrist	Algorithm (Sadeh)^e^	0.818	0.913	0.928	<0.001
	Decision tree^g^	0.943	0.999	0.980	

a - Area under the ROC curve calculated as sensitivity multiplied by specificity before data was rounded;

b - defined as the probability of correctly classifying bedtime rest period;

c - defined as a probability of correctly classifying activity period;

d - Wilcoxon signed rank test;

e - automated computer algorithm;

f - cut points were 20 counts/min (bedtime) and 500 counts/min (activity);

g - cut points were 250 counts/min (bedtime) and 3000 counts/min (activity).

## Discussion

In this study, we developed and validated a decision tree algorithm to separate bedtime rest and activity periods using waist or wrist accelerometer data (minute-by-minute counts) for 10–18 year-old youth. We showed that the proposed cut points for the accelerometer worn either on waist or wrist could provide >0.90 sensitivity and specificity for correct classification.

The need for establishing bedtime rest and activity cut points is underscored by a recent progress in accelerometry technology allowing wearing relatively nonintrusive monitors continuously for several days. For example, the National Health and Nutrition Survey (NHANES) is currently measuring physical activity using a wrist-mounted accelerometer worn for seven days and 24 hours per day.

The rationale for separating “bedtime rest” from “activity” is a need for categorizing of 24-h accelerometry data into the activity intensity categories. This approach is different from commonly used “wake hours” protocols in which participants are asked to wear a monitor from “waking up until going to bed, except during bathing, showering, and water-related activities.” The “wake hours” protocol approach is causing well-known concerns about adherence to the monitor wearing instructions and under or overestimating time spent in various intensity categories.

In the present study, we used a term “bedtime rest” to define periods of inactivity longer than 60 minutes and “activity” to define periods that activity above the established cut point was detected. In the current mainstream accelerometry literature, terms used for the “rest” category range from “sleep” to “sleep-period time” [33]. In addition, daytime rest (e.g., daytime naps) is usually classified as sedentary activity. We thought that “bedtime rest” could be a reasonable and at least temporary compromise between various terms used in the physical activity behavior literature. It would be beneficial if a consensus of the scientific community could be reached in the near future as it was with the term “sedentary behavior.”

A new challenge in the process of analyzing continuous accelerometry data (24 h) is to differentiate rest from sedentary behavior [34]. Modest improvement in the accuracy of bedtime rest versus sedentary behavior classification has the potential to improve our knowledge of the relationships between PA, PA-induced energy expenditure, and related health outcomes. Thus, the proposed approach might be especially important in population-based studies in which the sedentary behaviors and sleep patterns are often linked to health consequences and longitudinal risks for chronic diseases.

In this study, we used a decision tree approach to separate activity periods from bedtime rest periods, and the ROC approach for determining cut points that yielded the least misclassifications of bedtime rest. The ROC curves that generated an optimal bedtime rest cut points ranged from 20 to 30 counts/min for waist and 250 to 300 counts/min for wrist. The selected optimal cut points with the highest AUC were 20 counts/min for waist and 250 counts/min for wrist. The selected optimal cut points for the activity with the highest AUC were 500 counts/min for waist and 2,500 counts/min for wrist. The performance of the classification model was similar between the development and validation sets.

The study has several strengths. The room calorimeter data allowed us to discriminate between bedtime rest and - activity including sedentary behavior using objective measurements of EE and mechanical work. We used a relatively large group (n = 81) of male and female youth 10 to 17 years old with BMI ranging from16 to 40 kg/m^2^. Random selection of the development and validation sets allowed a robust performance of the model. We did not find significant differences in criteria for the cut points' selection between males and females. Although the differences between males and females may emerge in larger studies, our results do not suggest a need for gender-specific algorithms in youth. However, it has been recognized that children and adolescents have different patterns of physical activity than adults (e.g. short bursts of vigorous activity). It has been shown also that age-specific algorithms might result in more accurate assessment of physical activity than equations/algorithms not using age as a factor.

The study has some limitations. We used a uniaxial Actigraph that has been recently replaced by the triaxial models. However, it has been documented that, despite apparent differences between the uniaxial and triaxial accelerometers in the total number of counts for some vigorous activities, differences did not impact the activity intensity classification and indicated that the monitors were comparable when assessing sedentary behavior in youth [35] and older adults [36]. Applicability of our decision tree to other accelerometers (e.g. Actical, RT3) should be tested in future studies. The standardization of activity bouts performed in a room calorimeter may not accurately represent individuals' habitual daily PA patterns. Youths usually perform mixed and combined movements in their normal activities of daily living and different characteristics of the same activity can be observed from the same person. This could include variations in sleep patterns, rest and some sedentary behaviors, causing misclassification of bedtime rest as activity.

The accuracy of the decision tree could be likely improved by using an automated search for thresholds and cut points. The manual method we used integrated a combination of rounded values from a range expected for bedtime rest and activity. It is possible we could have achieved greater accuracy by using non-rounded values, but the accuracy would not likely have practical significance (e.g. 47 vs. 50 counts/min). The manuscript presents a sample of the most relevant combinations tested (Table 2) and a larger dataset is available in Supplemental data available upon request.

The bedtime rest cut-point was set initially at 50 counts/min. We have chosen this value because it is an established cut point for rest/inactivity for children and adults [31], [35]. An examination of the data around marked transitions from activity to bedtime rest confirmed that epochs below 50 counts/min were predominantly associated with bedtime rest (data not shown).

The decision tree developed in this study did not attempt to assess sleep, which is most likely included in the bedtime rest category. The sleep assessment requires a specific measurement approach and/or algorithm. First automated sleep scoring algorithm was developed by Sadeh and colleagues [18]. The Sadeh's algorithm was recently used for estimating sleep time in free-living children wearing waist-warn accelerometer and the results were compared with results from a wrist-worn accelerometer [19]. However, the algorithm required a diary to evidence the bed and wake time intervals and individual manual replacement of data-point by researcher's estimates drawn from raw data [19]. Our decision tree does not require either diary or any manual replacement of data. Nevertheless, future research using data from accelerometers worn for 24 h/day and several days should test the decision tree in other age groups and validate it against polysomnography. Any comparison is likely to be imperfect, however, because polysomnography provides estimates of sleep that go beyond inferring rest and activity patterns from accelerometer movement data [37]. The advantages of accelerometry over polysomnography for many clinical and epidemiologic studies include its convenience and the ability to record activity in free-living. In this study, relative to room calorimeter minute-by-minute data, the Sadeh's algorithm overestimated rest period and included potential sleep episodes outside of the bedtime rest occurring mostly during daytime hours.

Data were collected for approximately 24 hours and longer observation periods such as an additional day would provide more data from daytime activities. However, the feasibility of conducting studies in the room calorimeter for a period longer than 24-h is extremely difficult in youth. Nevertheless, longer (e.g. 7-day) studies in the free-living environment usually require self-reported bedtime rest and activity time markers obtained typically using a self-reported measure that is likely less objective and precise than a room calorimeter.

Physical activity estimates by the decision tree had a level of error much lower than that considered clinically irrelevant (i.e., 10%) in physical activity measurement studies [38]. However, there is no standard established to facilitate interpretation of the accuracy when estimating bedtime, wake/activity time, or sleep-period time with accelerometry. In our decision tree, periods of bedtime rest shorter than 60 min between beginning and end of the period were categorized as “activity.” This approach could cause that some periods with very low activity level (e.g., daytime naps) will be classified as “activity” and in further analysis most likely as a “sedentary” intensity category.

Despite these limitations, our decision tree can be used to classify bedtime rest and activity in studies larger than the present work and to rank the youth according to time spent in physical activity categories. Obtaining such data has relevance to obesity and metabolic syndrome among other health concerns, and therefore it is of significant clinical importance to youth's health [39]

In conclusion, we found that the decision tree developed for a waist- and a wrist-worn uniaxial accelerometer has good power for accurate separation of time spent in bedtime rest from activity in youth. Relative to the room calorimeter analysis of the same data, the decision tree yielded an estimate that was comparably precise. Application of the decision tree algorithms in population-based studies may lead to better prediction of time spent in bedtime rest apart from sedentary and active behaviors. More research should be conducted to verify our decision tree and/or optimize in studies with larger sample sizes and across settings with different populations.
